# An Historical Account of the More Important Operations in Surgery with Especial Reference to the Mode Employed by Dr. Von Wattmann

**Published:** 1843-07

**Authors:** 


### ART. III.

*Geschichtliche Darstellung der grösseren chirurgischen Operationen mit besonderer Rucksicht auf Edlen von Wattmann's Operations-Methoden.* Von Dr. FERDINAND HEBRA.—*Wien*, 1842. 8vo, pp. 434.

*An Historical Account of the more important Operations in Surgery, with especial reference to the mode employed by Dr. Von Wattmann.* By FERDINAND HEBRA, M.D.—*Vienna*, 1842.

THIS work is written as a manual for the use of the students at Vienna and the German universities, and contains a series of articles devoted to the history and progress of operative surgery, with a review of the present practice on the continent and in this country. To the end of the various articles is added an especial account of the practice of Dr. Von Wattmann, for the convenience of the students of Vienna, as well as on account of the respect of the author for the skill and kindness of his teacher.

The work is written with great care, and evidently with great attention to the works of the old writers. The work of Sprengel is taken as the basis of the historical part, whilst the standard of German surgery is deduced from the labours of Rust, Chelius, Dieffenbach, and Langenbeck. In France and this country the works of the writers during the last thirty years have been carefully studied, and their contents arranged with those collected from the German works, so as to form a complete and very honest summary of the present condition of operative surgery.

The history of surgery is more generally studied in Germany than in this country, and appears from Dr. Hebra's account to form a part of the examination at Vienna. The medical literature of this country is deficient in any good history of the progress of surgery. To the few who wish to study it, the works of Portal* in France, with Sprengel† and Zang‡ in Germany, afford a basis for further study. To the majority of readers, each surgical operation is only regarded for its own peculiar merit, without reference to anything else; and in the writings of most authors on surgery, the operations of Cheselden and Dupuytren, Taliacotius and Dieffenbach, are all classed together, without the slightest respect for their antiquity or any mention of their authors; the operation itself and not its author being the point always kept in view.

The present article contains an abstract of the work of Dr. Hebra, with a more complete summary of the practice in this country, as deduced from observation and a study of our surgical works. The principal points in the historical part, which are connected with the present mode of operating, are touched on, as being of sufficient interest in themselves, as well as deserving of mention, from respect to the originators of them.

1. *Trephining.* Perforation of the skull for the evacuation of blood, or for the relief of insensibility or vomiting after injuries of the head, appears to have been performed as early as 400 B.C., by Hippocrates himself, or one of the same family. This operation, however, was either performed with an instrument resembling a large gimlet, or with a circular saw, like the modern trephine, worked with a wimble or brace, and requiring the use of both hands. With the former instrument a simple perforation was made, whilst with the latter a circular portion of bone was removed. The danger of wounding the brain or membranes being great when the simple borer was used, the perforation was not generally made quite through the skull, but only partially; a thin portion being left to separate by exfoliation, and the relief being thus necessarily postponed till this event occurred. When simple fissures or cracks existed, a black fluid was rubbed on the bone, which sinking into the fissure, enabled the operator to trace it; this part and the bone round it were then removed with a kind of rasp, if the fissure was only shallow; if it extended deep, the trepan was employed. The trepanning instruments at this time consisted of the perforator, the trepan worked by the brace, and the rasp, to which we find added in the time of Celsus, or about the first century of the Christian era, the hammer and chisel, to remove portions of bone between two or more perforations; and the *meningophylax*, an instrument resembling a small mason's trowel, the metallic

* Portal. Histoire de l'Anatomie et de la Chirurgie, 1770. Seven Vols. 8vo.

† Sprengel. Geschichte der Chirurgie. 2 Bande, 8vo.—Halle, 1805, 1819.

‡ Zang. Darstellung blutiger heilkunstlerischer Operationen als Leitfaden, 1825. Five Vols. 8vo.

part of which was passed between the skull and membranes, to prevent injury to the latter from the slipping of the instruments.

The trepan worked with the brace appears occasionally to have injured the brain, and produced serious results; to avoid this result, we find that about this period a ring was added at a short distance above the cutting edge, or that three or four metallic knobs were here placed. These instruments were called by Galen (131 A.D.) *abaptista,* and held by him in great contempt; the chief instruments used by him being a pair of strong forceps to extract the loose portions of bone, and a strong cutting chisel or scoop to remove other portions.

From the time of Galen to 1363, the trepanning instruments were rarely used, the knowledge of injuries of the head decreased, and the medical plan of treatment was chiefly practised. Amongst the Arabians, the perforator and chisel were occasionally used; whilst in the west of Europe, the monks, who had become the surgeons of the age, treated these injuries with salves and powders. Little, however, as these monks benefited surgery, they appear not to have under-estimated their own labours, for they established a regular scale of payment in cases of injury to the head. The sum paid was proportionate to the size of the exfoliated portion of bone, and the noise produced by dropping it into an empty copper vessel. The knowledge of injuries of the head had now grievously decreased; thus Roger of Parma, (1206,) considered the non-passage of air through fissures of the skull, when the patient held his breath, as a means of diagnosis; whilst Lanfranchi of Paris, (1295,) considered the existence of fracture of the skull as detectible by the resonance of the head, when struck with a stick, and the inability of the patient to chew his food. Amidst this mass of ignorance we, however, find Guy de Chauliac, physician to Pope Clement VI., who introduced the trepan and added the central pin to it; he also employed instruments more cautiously than his predecessors, and limited their application to the more severe forms of injury of the head, and to those parts of the skull which are destitute of sutures.

A brighter era now, however, commenced in the operative and other departments of surgery, and the treatment of injuries of the head began to assume those features which it has since retained. Within a short period we find Andreas da Croce at Vienna, Ambrose Paré, with his indefatigable pupil, Jacque Guillemeau, at Paris, and Fabricius ab Acquapendente at Padua, all labouring at the improvement of practical surgery, the perfecting of their instruments, and the instruction of others by their well-known works. Up to this period, namely about 1550-1600, the trepan or circular saw worked with a wimble had been used. Fabricius ab Acquapendente now introduced the modern trephine, or circular saw worked with a handle, which required only the use of one hand. This instrument was, however, armed with prominences, till Andreas da Croce improved it, and described the trephine as used at the present day, without prominences or guard, but depending alone for its safety on the skill of the operator. Amongst the instruments used at the end of the sixteenth century, we may therefore enumerate the trepan, or circular saw worked with a wimble, the trephine, or circular saw worked with one hand, with or without central pin, elevators, chisels, the forceps, rasps, and strong chisels, acting on their lateral edges, with various instruments

LIBRARY OF THE

Orleans Parish Medical Society for smoothing the edges of the cut and cracked bone. In addition to these, the Hey's saw deserves especial mention, as it is so frequently used in this country, in comparison with the various forms of round saws. This instrument, consisting of a small flat saw, with the teeth either in a straight or convex line, and fixed on a small handle, has been ascribed to Scultetus of Ulm, and is figured by him in the Armamentarium Chirurgicum. Scultetus, however, describes this instrument as used by others, and lays no claim to its invention, but only to the introduction of the "serrula versatilis," which is a complicated instrument, with a double set of wheels, and resembling in principle the modern "scie à molette" of Charriere at Paris. The Hey's saw is an instrument of much greater antiquity, and appears, from the account of Andreas da Croce, to have originated with Avicenna (974 A.D.), although some slight doubt exists whether Hippocrates himself was not to a certain degree acquainted with it.

The present surgical instruments for injuries of the head thus existed at a comparatively early period, but they form a part only of the number, as many surgeons added to and altered the instruments in a useless and frequently ridiculous manner; and it was not till the beginning of the eighteenth century that Mr. Cheselden, of St. Thomas's Hospital, and Mr. Sharp, of Guy's Hospital, simplified the instruments, and brought the trephine into more general use, which latter instrument Mr. Pott improved by substituting a wooden for an iron handle. Mr. Sharp practised the incision of the dura mater, when blood was effused under it, an operation which appears to have been practised as early as 1619 A.D., by Glandorp, surgeon at Bremen on the Weser. The operation of trephining over the sinuses appears for many years to have been dreaded as dangerous, till Hoffmann, of Altorf, (1653 A.D.) and subsequently, amongst others, Mr. Warner, of Guy's Hospital, trephined over the lambdoid suture, whilst Lassus, of Paris, (1783,) opened the longitudinal sinus without any bad result.

It is remarkable that the trephine should be still used in England, whilst the trepan or saw worked by the wimble is the instrument chiefly employed in Paris and Vienna. In the great majority of cases, however, in England, as the operation is performed for injuries accompanied with depression of bone, the Hey's saw is principally used. The chief advantage of the trepan is that it can be used by the most clumsy person, and requires very little delicacy of hand. The trephine, however, in the hands of a dexterous surgeon can be used with the greatest safety, whilst to a delicate hand a more complete sensation of the nature of the parts cut through is communicated to the hand, and not unfrequently, by the slight pressure of the hand the internal table is cracked and not sawn, by which means the dura mater is less exposed to injury.

In this country, the exposed dura mater is treated as mildly as possible with a soft poultice or water dressing. In the account of the practice of the Vienna school, Dr. Hebra mentions that unless circumstances exist to contraindicate it, the portion of bone, which has been sawn out, is inserted again in its old position in the opening made by the trephine, and covered again with the integuments and other parts, which are united by suture or plaster, except at a small spot, which is left open for the evacuation of pus or other fluids. If, however, in a few days the replaced portion shows no inclination to unite with the neighbouring bone, and assumes an unhealthy colour, it is removed, and proper dressings are applied.

2. *Harelip.* It is remarkable that so common a deformity as harelip was not relieved by operation till a comparatively late period. The operation for the removal of harelip is described by Celsus; but even the remarks made by this author are less full than might be expected, and simply to the effect, that the best mode of operating in cases of simple fissure consisted in making an incision on the convex edges of the fissure, whilst in the double fissure he appears to have removed the central portion, and inserted a piece of skin from the neighbouring parts. Abul Kasem, an Arabian physician, resident in Spain, (1122,) adopted the practice of applying the actual cautery to the edges, and uniting them with plaster, which operation appears to have been the general practice, till Ambrose Paré (1509) adopted a modification of the plan now generally used. This experienced surgeon, in cases of harelip and wounds resembling it, pared the edges of the wound, and then united them, by passing needles through the lip, which were more firmly fixed by thread twisted round the needles in the figure of eight, the end of the thread, however, being passed through the eye of the needle. This mode of fastening the thread was copied from the manner in which the ladies and tailors of the day wound the thread round the needle, and thus carried both safely in their cuffs or caps. This operation of Ambrose Paré was improved by Fabricius ab Acquapendente, (1557,) who suggested the advantages derived from making an incision of the mucous membrane, which united the lip to the gum. The generation of surgeons succeeding these two appear to have displayed their ingenuity in trifling alterations; some used metal pins with the ends cut off, whilst others put pads of lint under their extremities; some used triangular needles, whilst others used very small sewing needles, so small, indeed, that Heister bestowed on the profession a needle-holder. At last Petit, of the Academy of Surgery, (1674,) introduced the silver pins with moveable steel points, which were not only more convenient to use, but did not rust or stick in the wound.

CHARITY HOSPITAL, MEDICAL LIBRARY.

The mode of making the incision, the best form of needle, and the mode of applying the thread being thus settled, the next change in the operation was caused by the disputes concerning the relative advantages of sutures and needles, as compared with plaster; the majority, however, continued to use needles, employing frequently bandages and plaster in addition. The general object of these plasters was to press the parts of the face forwards, and render retraction of the sides less likely to occur. By some, bandages and plasters were carried round the back of the neck and over the lip, whilst others only applied them, as is frequently now done, to the cheeks. The instrument for holding the lip consisted of two blades working parallel, and of such relative dimensions, that the blade applied on the under side of the lip afforded a broad surface, made of wood or horn, for cutting the lip accurately upon; it appears to have been first introduced about 1801, by A. von Beinl, a military surgeon at Vienna.

The operation for harelip, as performed on the continent and in this country, differs little in the mode of incision and manner of bringing the parts into apposition. By some the double harelip is treated at once, according to the plan of Desault, whilst others prefer operating on one side first, generally commencing with the worst, and then treating the second side at a subsequent period. The choice, however, of this plan must depend on the nature of the fissure, as in cases of great separation the attempt to restore both sides at once would be very difficult. The knife-edged scissors are free from the objection, urged against common scissors, of crushing the lip, and are used by many of the principal surgeons at Paris as well as in London. The simple knife is, however, more generally used by the surgeons of Edinburgh, whilst at Vienna the lip is cut with a knife on a piece of wood or plate of horn placed under it. The harelip pins of Petit, although still frequently used, are not by some preferred to strong common needles or pins, united, however, generally with the twisted thread of Ambrose Paré, whose simple copy of the tailors' needle has not been yet improved on.

The practice of Dr. Von Wattmann does not appear to correspond in two very important points with that employed most frequently in this country, in reference to the proper periods for removing the pins, or the manner of feeding the child. The needles, according to Dr. Hebra, are not removed till the seventh or eighth day, whilst in this country the second, third, or fourth days are frequently chosen as the proper time for applying plaster or other means in the place of the needles; thereby lessening the chance of cicatrized dots in a part where any unnecessary blemish is much to be deprecated. The child is also recommended to be fed on artificial food, and by no means to suck; experience, however, points out the impropriety of such advice. If the pins are short and neatly arranged, the child may be allowed to take the breast from the first, and thus be kept quite quiet, thereby preventing the derangement of the health as well as the separation of the parts from crying.

3. *Tracheotomy and laryngotomy.* The operation of making an opening into the trachea appears to have been performed as early as 100 B.C., by Asklepiades, of Bithynia, and to have been followed with success. Aretæus, however, brought the operation into disuse, by stating that inflammation and spasm of the part, as well as the sense of suffocation, were increased by it, and that the wound did not heal up. In spite of these objections, Antyllus, a surgeon in Adrian's army, revived this operation in cases of difficult breathing from pressure of the parts in the fauces and the presence of a foreign body in the trachea, forbidding, however, its use where the lungs were affected. The operation performed by Antyllus consisted in an incision down to the trachea, of which he removed three or four rings. The fear of fistulous openings remaining was removed by Abul Kasem and Ebn Zohr, in Spain, the former of whom cited an instance where a wound of the trachea made by a maniac on herself had healed up, whilst the latter removed a portion of the trachea of a goat, and found that the opening closed readily.

The next improvement, introduced by Fabricius ab Acquapendente, consisted in reaching the trachea by a series of incisions, and inserting a tube into the opening which he had made. This tube was originally straight, but was curved by Julius Casserius (1545). The chief variations in the operations introduced by various writers consisted in the length of the external incision, and the oblique or vertical direction of the incision in the trachea. Frederick Dekker of Amsterdam, in 1694, introduced the plan of puncturing the trachea at once with a trocar and canula, and after withdrawing the trocar, leaving the canula in the opening. This operation was revived within the last few years by Mr. Wood, in a long and very laborious paper in the Medico-Chirurgical Transactions, who suggested the curved canula instead of Dekker's straight tube, armed with a jointed trocar or knife accurately fitting the canula. Desault is generally said to have first performed the operation of laryngotomy, and is certainly deserving of the credit of having first set the operation well before the profession; but he does not describe this operation as quite new, or claim the discovery of it. In common cases of difficulty of breathing, this able surgeon cut the crico-thyroid membrane, but when a foreign body existed in the trachea, he divided the anterior edge of the thyroid cartilage. This operation was modified by Jourdain, who cut the crico-thyroid membrane, and then divided the anterior part of the cricoid cartilage and the upper rings of the trachea. Since his period, surgeons have employed the incision of the crico-thyroid membrane extended upwards or downwards, so as to avoid the thyroid gland on the one hand, and the chordæ vocales on the other.

The operation of opening the larynx or trachea does not afford either room for much variety or generally much time for thought; the difficulties are great and variable, especially when the urgency of the case affords no time for delay. The trocars of Dekker and Wood are rarely used, and do not appear devoid of danger. If the trachea is exposed, these instruments might be used with benefit and safety, but if the trachea be punctured at once, when the parts are deep and the trachea small, this tube might even miss the trachea or wound its posterior wall, whilst any vessel, usual or unusual, from the thyroid veins to the arteria innominata, must run its chance. The knife is the especial instrument in the hands of the surgeon in this as in most other operations. The danger of blood flowing into the trachea has been variously estimated; by some it has been supposed not to be dangerous in moderate quantity, whilst by others it has been considered as the chief danger of the operation. It appears that in many cases no bad symptom has occurred from even considerable bleeding into the trachea; but the case of M. Roux, (Andral, Clinique Médicale, tome iv. p. 209,) with its excellent and ingenious treatment, affords such an instance of the danger from this occurrence as to serve as a warning for ever, and to justify direct reference to it here. M. Roux was performing the operation of tracheotomy. The trachea was just opened, when a small quantity of blood entered into it with the first rush of air, and prevented its entrance into the bronchial tubes. The patient became insensible, and the arteries ceased to pulsate, the face assumed the whiteness of death, and the pulsations of the heart were no longer detectible. Without the least delay, M. Roux introduced a gum elastic tube into the trachea, and by sucking the air through it drew out part of the blood. In a short time respiration was established, and the heart began to beat, consciousness, however, returned very slowly, and it was not till after two days that the patient first began to recognize the persons standing round her bed.

The old tubes are still frequently used; cases, however, often occur, in which so much irritation is produced by them, as to render them inapplicable. Under these circumstances the removal of a portion of the trachea has been practised; sometimes also the separation of the sides of the wound with a curved wire or other means has been found to be the most convenient course of proceeding.

4. *Œsophagotomy.* Although the possibility of this operation was mentioned by Verduc of Paris as early as 1611, it does not appear to have been performed till 1783, when Goursaud of Limousin extracted a bone from the œsophagus by cutting on the left side of the neck; this operation was successful, and was performed a second time by Roland shortly after.

Carlo Guattani, in 1772, wrote a very valuable paper in the Memoirs of the French Academy of Surgery, describing some experiments on dogs to show that wounds of this part healed readily, and that it could be easily reached in man by cutting between the trachea and left sterno-thyroid muscles. In 1779, Eckholdt of Leipsic suggested the propriety of reaching the œsophagus between the two origins of the sterno-mastoid. These two operations were so difficult that, till 1820, the attention of surgeons was chiefly directed to the removal of foreign bodies in the œsophagus by vomiting and instruments, and to this period we are chiefly indebted for the information on this subject which we possess. Vacca Berlinghieri of Florence, in 1780, suggested the best plan of reaching the œsophagus, by making an incision between the sterno-thyroid and sterno-hyoid muscles in front, and the sterno-mastoid behind, the omo-hyoid being cut across, and the œsophagus rendered prominent by the introduction of a bulbed steel catheter. The œsophagus was divided upon this and the obstacle drawn out with the forceps. The chief varieties in the operation have consisted more in the instruments used than in the situation; in the occasional cases which occur the situation selected by Vacca Berlinghieri being generally preferred. In this country the scalpel and forceps, with the occasional use of the bulbed sound or catheter to render the œsophagus prominent, have been generally used with a hook to hold the œsophagus and forceps to extract the obstruction; such also appears to be the practice at Vienna. Considerable variety, however, exists as to the instruments used for this operation at Paris. Thus M. Lisfranc introduces a curved canula, with a forked stylet, on which the œsophagus is divided, whilst MM. Roux and Velpeau prefer a canula with a spring blade, with which the œsophagus is divided from without inwards. The chief peculiarity of M. Begin's operation consists in cutting down to the œsophagus midway between the trachea and sterno-mastoid, and opening it without the use of any instrument except the simple scalpel.

5. *Paracentesis thoracis.* This operation appears to have been performed at a very early period, and is said to have been originally suggested by the recovery of a man after a wound of the chest, who, on the want of any relief to a disease of the lungs by medicine, had gone to battle in hopes of being killed. Such may or may not have been the case; but there is no doubt that this operation was performed at a very early period, as it is described very fully by Hippocrates, and some means of diagnosis given and indications for the place of puncture. The patient is directed to be shaken to make the fluid rattle, whilst a wetted cloth is recommended to be wrapt round the chest, and the spot which becomes dry first is to be chosen as the place of operation. If the case was one of hydrothorax, Hippocrates recommends that the rib should be perforated and the fluid partially evacuated, after which a plug is to be inserted and the fluid gradually drained off during the succeeding thirteen days; In empyema, however, the intercostal space was punctured with a lancet, and a certain portion let out, whilst the remainder was evacuated twice in the day for nine days, after which the opening was either allowed to close gradually or injections were made into the chest, according to the nature of the matter. Galen introduced the plan of trepanning the sternum in hydrothorax, and drawing out the fluid with a syringe; whilst the chief Arabian and Grecian surgeons used either the lancet-shaped instruments for perforating the intercostal muscles, or applied cauteries to the prominent part of the swelling, and practised the operation of perforating the rib very rarely. The experience of Ambrose Paré induced him to recommend this operation in cases of wounds of the chest and fractures of the ribs, as well as in empyema; whilst Fabricius ab Acquapendente also included cases of inflammation of the pleura, suppuration of the lung, internal abscesses, and wounds. The incision of the walls of the chest was practised by Fabricius ab Acquapendente, Diemerbroeck, and Solingen till 1694, when Vincent Drouin introduced the trocar for this operation.

The two operations of cutting down to the pleura and opening the chest with a knife or trocar have been employed by Delpech, Morand, Boyer, and Van Swieten, whilst the puncture of the chest with the trocar pushed right through has been employed by Sharp, Sir Charles Bell, and others in England; by Laennec, &c. in Paris; and by Schuh of Vienna; both operations being still practised and supported by strong authorities. By drawing down the skin a valvular opening may be made, either with the knife or trocar indifferently, and an equally free opening afforded for the canula; when, however, the least admission of air is objectionable, the introduction of the small trocar and canula affords an advantage over the knife, as, in addition to the valvular opening, the sides of the wound fit the canula more accurately, the flow of fluid can be graduated and intermitted at pleasure. If the wound is to be left open and the admission of air is immaterial, the opening made by the knife is more free than that made by the trocar, as the former is one clean cut, whilst the latter is formed by three small radiated incisions. The most prominent part of the chest will often point out the best situation for puncture; there are, however, some situations in which the opening can in general be more conveniently made than in others. The lower edge of the rib is to be avoided, on account of the intercostal artery; whilst the junction of the serratus magnus and obliquus externus abdominis is also objectionable on account of the small arteries and nerves which are usually situated there. Professor Leber stated that a convenient spot might be found, by passing a line horizontally round one side of the chest, from the ensiform cartilage to the spine, and choosing the spot at the junction of the middle and posterior thirds for the seat of puncture. This line would fall between the seventh and eighth ribs, and is the situation preferred now by Von Wattmann of Vienna, and formerly by Walther of Halle. Now, although this situation is supported by such good authority, and even a lower situation has been recommended by Pelletan, Sabatier, Larrey, and Desault, and is still practised in France, yet a higher situation is generally preferred in this country on account of the danger of wounding the diaphragm, lung, or liver. The space between the fifth and sixth ribs has been recommended by Hey and Laennec, and that between the sixth and seventh by Sharp, Bromfield, Sir Charles Bell, S. Cooper, Callisen, Rust, and Von Graefe, either of which is indifferently preferred by the principal surgeons of this country. Before leaving this subject it is right to mention the fine grooved needle, which has been introduced for puncturing the chest in obscure cases, and for the gradual removal of the fluid, by the late Dr. Davies of the London Hospital, but which has been brought more fully before the profession by Dr. G. Babington of Guy's Hospital; to which may also be added the double trocar of Dr. Skoda and Dr. Schuh of Vienna, by which the entrance of air into the cavity of the chest is effectually prevented.

6. *Paracentesis abdominis.* This operation was performed as early as 1100 B.C., and appears to have been remarkably fatal till the time of Ambrose Paré, who recommended the slow evacuation of the fluid, the careful bandaging of the patient, and the accurate closure of the wound. This operation was generally performed either with a pointed instrument, knife, or caustic, until 1626, when Sanctorius Sanctorinus introduced the trocar and canula into general practice, which had really been invented by Girault, a French surgeon some time before. The trocar of Sanctorius was a round pointed instrument encased in a canula, with which he punctured the umbilicus in men and the vagina in women, under the bedclothes and out of the sight of the attendants. This canula of Sanctorius was altered and modified in various ways without much improvement by many; thus L. Petit added a fissure in the canula, which allowed the edge of a knife to be passed through to enlarge the opening, whilst Andrée introduced the flat trocar with the lancet point, to which a canula, made of two semicircles of tube screwed together, was fixed. This canula was flattened by the trocar extending it laterally, but on the withdrawal of the trocar assumed an oval or round shape. This trocar was subsequently altered for a round one by Mr. Wilson of Glasgow. The lancet-pointed trocar, with a flat or round stem, as well as the simple round trocar, with a round point, had now been all introduced, when John Aug. Ehrlich of Leipzic, in 1760, introduced the trocar with an enlarged extremity and canula, the latter accurately fitting the part behind the enlargement of the trocar, and divided into three parts at its end, to allow the easy withdrawal of the trocar. This is the instrument now generally used, whilst the same modification has been applied to the flat trocar. Injections of fluid into the abdomen were tried at various periods and continued by some down to the end of the last century. Thus Mr. Warrick of Truro, in 1744, after drawing off twenty pints of fluid from the abdomen of a woman of fifty years of age labouring under ascites, injected several pints of a mixture of claret and Bristol water without any very bad effect, and thus cured the dropsy. Since this period, injections have been recommended by some and practised by others; those of aromatics by Heuermann of Copenhagen; of lime-water by Weitz; and barley-water by Chopart, Desault, and Bertrandi. Two cases are recorded by Mr. S. Cooper, where ovarian cysts were injected by Mr. Ramsden with a fatal result.

The abdomen has been punctured from the rectum, vagina, and scrotum by various surgeons, but the parietes of the abdomen have become now the common situation of puncture. The practice at Vienna is to puncture the umbilicus, or the lower part of the abdomen, on the right or left side, at a point midway between the anterior superior spine of the ilium and umbilicus. The umbilicus has been recommended by Ollivier and Bigot, of Angiers, whilst M. Velpeau recommends the lateral part of the left side. The puncture of the umbilicus has become very rare in England, and is not practised, rather from want of any reason for it than from any objection against it; whilst the puncture midway between the anterior superior spine of the ilium and the umbilicus is not practised on account of the danger of wounding the epigastric artery, which is occasionally carried out to one side with the flattened rectus, whilst at other times it retains its natural situation. On these accounts the puncture of the abdomen is generally made in or close to the median line, by which means all arteries are avoided, and only in very rare cases a vein of any size is exposed to injury.

7. *Catheterism.* Catheters appear to have been used frequently at the time of Celsus, of a curved form, and from nine to twelve or fifteen inches long for men, whilst somewhat similar instruments, but from six to nine inches long, were employed for women. Amongst the ruins of Pompeii, some long straight iron tubes were found, which appeared decidedly to be instruments for passing into the bladder. Catheters appear to have been regularly used from their first introduction, and amongst a rich people addicted to rich living and sensual pleasures must have been often required; the passing of the catheter at last became an art, and when the itinerant lithotomists travelled about the country, assumed a supposed degree of perfection. These men treated difficult cases with a boldness which could only be based on some experience of good, and tried to extol the difficulties of everything that was comparatively easy: they passed the catheter with a facility which was acquired by constant experience, and with a dexterity and rapidity of motion, which astonished the spectators, and received the well-known name of "tour de maître."

At the end of the sixteenth century flexible catheters of leather and horn were introduced, which were convenient, but far inferior to the modern elastic catheter. It is not clear to whom the merit of originating the modern elastic catheters is due. Theden, Pichel of Wurzbourg, and Bernard of Paris, are mentioned amongst the introducers of them. The elastic catheters for a considerable period were made of silver wire, twisted in a spiral form and covered with elastic web; these were found very liable to corrode and break, and the introduction of the common elastic gum catheter as at present used was a great addition to surgery. These were imported for a very considerable period from Paris, but are now made to a considerable extent in this country, of the most varied sizes and forms. In affections of the prostate gland as well as in cases where the catheter is required to be left in the bladder, these instruments are of the greatest use, whilst their employment has increased latterly in cases of stricture since their recommendation as well as improvement in the adaptation of suitable handles by Sir B. Brodie.

The utility of straight catheters was pointed out by Montagu, Lieutand and Gruithuisen, but did not excite much notice in the medical profession till Amussat and Civiale devoted their attention to this subject. On the continent and especially at Paris, this plan has attracted more notice than in this country, where the plans of our forefathers meet with more respect, and any new remedy must possess some superiority before we lay aside our hereditary practice. Catheters undergo less change than any other instruments. The most convenient metal is found to be silver, and as the urethra possesses a definite length and breadth, the powers of invention are limited. The long prostate catheter of Sir Astley Cooper, the short sharply-curved catheters of Sir Benjamin Brodie with their solid firm handles, and the catheters suited to the natural curve of the urethra introduced for the operation of lithotrity by Baron Heurteloup, and for common use by Mr. Stanley, are the principal instruments used in this country in addition to the silver catheters of the ordinary curve, the real excellence of which it has been universally agreed, even by the inventors, exists only when used by the good anatomist as well as experienced surgeons.

8. *Hernia.* The operation for the relief of strangulated hernia was evidently known to Celsus in an imperfect manner, but appears to have become to a certain degree obsolete after his death till the time of Ambrose Paré and Franco, the former of whom appears to have relieved cases of inguinal hernia, by opening the sac and cutting out a small portion at its neck. It does not appear, however, to have been decided till the end of the sixteenth century, in what situation inguinal hernia was generally strangulated, when Abr. Cyprian in Holland, laid down the fact, that the strangulation was generally caused by the parts at the abdominal ring. To this fact very great additions were made by Haller, Mery, Pott, and the two Hunters, who described more accurately the anatomy and pathology of inguinal hernia, as well as the peculiar nature of the congenital rupture.

Louis Petit appears to have practised the operation of dividing the stricture outside the sac as early as 1720, which operation was supported and performed by Monro Primus, about fifty years later. This operation has been variously estimated by the writers and surgeons of the present day, especially Mr. Key, who has been its zealous supporter; whilst its value has been discussed in all the modern works on this subject, more especially those of Professor Richter and Mr. Lawrence, as well as illustrated by cases from the practice of Mr. Luke, Mr. Lloyd, and Mr. Liston. No subject in the whole of surgery has been rendered more complete than that of hernia within the few last years, whether we consider the anatomy of the parts, the operation itself, or the medical treatment. In each country of Europe, where medicine has been regularly studied, this subject has attracted the attention of the best men in the profession. In Italy Scarpa, in Germany Richter and Langenbeck, in France Dupuytren and Jules Cloquet, in England Percival Pott, Sir Astley Cooper, and Mr. Lawrence, have all made it the subject of exclusive and laborious essays and works. The important parts of this operation have become thus so definitely settled, and the reasons for them so evident, that the history of this operation presents matters of less general interest than that of any surgical operation, if we consider its gradual progress; it is, however, more remarkable than any other for the early period at which many of the most important points of its treatment were decided.

9. *Lithotomy.* The operation for stone appears to have been performed as early as 318 B.C. at least, and to have consisted in cutting down on the stone through the perineum, which was rendered prominent and tense by the pressure of the stone, which was pressed against the lower part of the bladder by the introduction of the finger into the rectum. This operation was called the operation by cutting on the gripe or the lesser apparatus, and was performed by Celsus, the surgeons of Alexandria, Guy de Chauliac, and several itinerant lithotomists down to the sixteenth century. Lithotomy in women was performed by Celsus, by making an incision between the urethra and pubis, whilst in virgins the incision was made on the outer and lower part of the left labium. In 1525, Johannes de Romanis of Cremona introduced the plan of making an incision into the bulb on a sound, this incision being then continued into the membranous portion of the urethra only for a short distance. The urethra and neck of the bladder were then dilated with two "conductors" fitting into each other, on the withdrawal of which, the "forceps" and "latera" were introduced, and the stone removed. This operation was continued more or less till 1697, and was practised by the principal continental surgeons. The operation from its complexity was named the "apparatus major," as well as the Marian operation, from a wonderful account of it published by Marianus Santo da Barletta, who described the instruments and operation in such flowing and eloquent language, that his name has been transmitted to posterity with the operation.

It is said that Hippocrates bound his pupils by an oath not to perform lithotomy, and if we refer to the accounts of the Marian operation, we might be tempted to agree in the propriety of his decision: whether we consider the parts divided, or the extreme dilatation of the membranes and prostatic portions of the urethra, the operation has nothing to recommend it, and is far inferior in excellence even to the old Celsian method or operation by the gripe. With all the skill of the first surgeons in Paris, the deaths from this operation amounted to more than 50 per cent., whilst imperfect cures and fistulous passages also occasionally occurred after the operation. This operation was unfortunately perpetuated by an accidental circumstance. Lawrence Collot learnt it from one of the pupils of Romanis, and practising it at Paris was made Lithotomist Royal, an office which continued in the same family for about 150 years, whilst the very worst part of the operation, the extreme dilatation, was held forth by Collot himself as the whole secret of the proceeding.

We now come to one of the most remarkable circumstances in the whole history of surgery, whether we regard the complete change in the mode of operation, its mode of origin, or the conduct of the originator himself.

In 1697, Jaques Beaulieu, commonly called Frère Jacques, appeared at Paris, stating that he was inspired from heaven to cut for stone, fistula, and rupture. He claimed neither money nor reward for his operation; (which he had secretly learnt from an itinerant lithotomist of the name of Paulani;) he lived a life of the greatest practical piety and self-denial, and only asked them to allow him enough to obtain the common necessaries of life, and to keep his instruments clean. His operation consisted in passing a long dagger-shaped knife from the left side of the perineum by the tuber ischii right into the bladder, which was rendered fixed by a round large sound with a short curve passed through the urethra. The bladder was thus wounded in its lower and back part, and this wound was continued forwards and enlarged by cutting towards him. He then withdrew his instruments, and guiding his forceps with a conductor or finger, pulled out the stone. Thus this uneducated friar changed the operation for stone from a dilating to a cutting operation, and without even a pretence of scientific knowledge saved as many, perhaps even more patients, than the best operators of the day. This man made, however, but one friend, and was at last obliged to leave Paris in 1698, to travel in Germany and Holland; but in 1700 his only friend, Fagon, the royal physician, became a sufferer from stone, and recalled him to Versailles, where he studied anatomy under Du Verney, and learnt his new operation. This new operation consisted in passing a grooved staff into the urethra, upon the prominent part of which an incision was made on the left side of the perineum through the transversus perinæi and levator ani muscles; he then carried his knife, an ordinary scalpel, along the groove through the prostate and neck of the bladder, and extracted the stone with the forceps. This operation with the improvements collected during 140 years, we now retain as the best lateral operation; yet it benefited Frère Jacques but little. He practised at Paris, and the greatest capitals of Europe, taught Marèchal and Raw how to perform his operation, and died in comparative poverty at his native village about the year 1714. The little money which he had acquired he gave to his relations; the medal bearing the motto "Ob cives servatos," presented to him by the senate of Amsterdam, was never found; and the golden sounds which had been also presented to him at the Hague, were well known to have been melted down before his death.

On the retirement of Frère Jacques, Raw acquired that great fame, which has transmitted his name to posterity. This famous lithotomist had seen Frère Jacques operate both before and after his improvement, and was supplied with bodies at Amsterdam for his anatomical lectures, whilst Frère Jacques was operating there. There appears to be no doubt that his secret was the poor friar's operation, by which this shrewd surgeon made a large fortune, and which he practised with the greatest success. He explained the operation to nobody, and even advised them not to believe what he said, and only gave them a slight hope of the revelation of his secret on his death. This secret never transpired, but what was more unfortunate a wrong idea of his operation became prevalent after his death, and misled amongst others Cheselden himself, who believed the accounts which Albinus, the pupil of Raw, gave of his patron's operation.

Cheselden, then surgeon at St. Thomas's Hospital, was willing to practise anything, which he could learn to be of use for the relief of others, even though it was unintelligible to himself; he therefore cut deep into the body of the bladder according to Raw's supposed plan, and met with the greatest disappointment. He then began to think for himself, and planned the lateral operation, closely resembling the operation of Frère Jacques, and in all probability the real operation of Raw.

The real operation of Cheselden has generally been copied from Dr.

Douglass' statement, and a wrong conception of its nature thus been formed. The latest form of operation, though certainly different from Dr. Douglass' description, is not very clear even in Cheselden's own words. In 1730, Cheselden, in an appendix to the fourth edition of his Anatomy, described the latter part of the operation thus : " I then feel for the staff, and cut upon it the length of the prostate gland, straight on to the bladder, holding down the gut all the while with one or two fingers of my left hand." This accords with the account of Le Dran and Morand, the former publishing in 1730, the latter in 1731 ; the former, however, is more particular, and mentions that after the point of the knife was inserted in the groove of the staff, that it was carried on in the groove to the end of the staff. The operation of Cheselden may thus be stated, on the authority of Cheselden's Appendix of 1730, and Morand's account in the memoirs of the Royal Academy of Sciences in 1731, and Ledran's account in his Paralèlles des Tailles in 1730, to have consisted in cutting between the accelerator urinæ and erector penis and by the side of the intestinum rectum ; the staff was then cut upon in the membranous part of the urethra, and the point of the knife inserted in the groove, which knife was then carried on through the prostate into the bladder, all the way running in the groove. The gorget was then passed along the groove of the staff and the forceps over it.

But here comes a question : Dr. Douglass, in his appendix to the History of the Lateral Operation, states that Mr. Cheselden's knife first entered the groove through the sides of the bladder immediately above the prostate, and afterwards the point of it continuing to run in the same groove in a direction downwards or towards himself, the prostate and muscular part of the urethra were divided. This account of Dr. Douglass appeared in 1731, he also adds the following words : " When he first began to practise this method, Cheselden cut the very same parts the contrary way ; that is, his knife entered first the muscular part of the urethra, which he divided laterally from the pendulous part of its bulb to the apex or first point of the prostate gland, and from thence directed his knife upward and backward all the way into the bladder, as we may read in the appendix he lately published to the fourth edition of his Anatomy. But some time after he observed that in that manner of cutting, the bulb of the urethra lay too much in the way ; the groove of the staff was not so easily found, and the intestinum rectum was more in danger of being wounded." In short Dr. Douglass states that Cheselden subsequent to 1730 pierced the bladder behind the prostate, and cut from behind forwards. The point therefore remaining to be decided is, whether Cheselden describes a different operation in his work subsequent to 1730.

There are two editions published during Cheselden's life subsequent to 1730 ; one in 1740, and another in 1750, containing an account of this operation, and both these describe the operation in the very same way as the appendix of 1730, but with an important alteration in a few words. The words " cut upon it (the staff) in that part of the urethra which lies beyond the corpora cavernosa urethra and in the prostate gland, cutting from below upwards to avoid wounding the gut," are substituted in the editions of 1740 and 1750 for the words " cut upon it (the staff) the length of the prostate gland straight on to the bladder,"

which are the words of the edition of 1730. This alteration "from below upwards" agrees with the account given by Dr. Douglass of the direction of the incision made by Cheselden latterly, the great mistake of Dr. Douglass appearing to consist in the point at which he supposed the incision to have been begun. The parts divided in Cheselden's operations appearing both before and subsequent to 1730 to have been the same, and the only difference in the operation to have consisted in the fact, that Cheselden divided the prostate down on to the staff, instead of cutting it from without inwards towards the rectum.

Claude Nicolas Le Cat had seen Morand perform Cheselden's operation, but subsequently introduced a modification of it, which was performed with a multiplicity of instruments, and had the disadvantage of making an opening of very uncertain size into the bladder, whilst Frère Côme attained to the same uncertain end with his lithotome caché, an instrument with a spring blade capable of making a very extensive incision.

The gorget with a cutting edge was introduced by Sir Cæsar Hawkins of St. George's Hospital in 1753, and has been modified chiefly by Scarpa and Mr. Abernethy, whilst its use has been recommended and practised by successive surgeons of the British metropolis. At the present day there are few hospital surgeons, who use it as the regular instrument, but by most its use is recommended under particular circumstances, and in some cases, as when the perineum is deep, the prostate much enlarged, or the parts in the perineum much diseased; whilst in some cases where the perineum is deep and large, the double cutting gorget is still used. It may, however be said, that the knife or simple large scalpel is the instrument generally used for lithotomy by the chief surgeons. Mr. Blizard's knife is also used at some institutions. The blunt gorget is still used as the most efficient dilator of the prostate, whilst the cutting gorget is still sufficiently often used to justify its insertion in the operating cases, but known to be an instrument of so great good or evil, that its recommendation is always accompanied with allusion to the accidents, too frequently fatal, occasionally arising from its use. In different countries, and even in different hospitals of the same town, various knives and staffs of different sizes and curves have been introduced, but the principle of operation has varied much less; the operation of Cheselden, with slight variation, is performed by the principal surgeons in this country, by Lisfranc in France, and by Dieffenbach and Von Wattmann in Germany. The lithotome of Dupuytren, recommended by his practice, and left as a legacy to Sanson on his death, still retains its place in France, though modified somewhat, and occasionally employed in conjunction with the knife. It would be difficult to mention all the modes in which the bladder has been reached by high incisions as well as by the rectum, but the perfection to which these have been brought would not be very interesting in a country, where the unanimous voice of the profession has rejected them, and when each successive year limits the modes of performance as well as the frequency of lithotomy.

Within the last few years the attention of surgeons has been chiefly directed to the study of the various conditions of health existing in persons, who are unfortunate enough to require lithotomy, whilst the operation of removing the stone without incision has become more generally practised by the profession. This is remarkably contrasted with the studies of the older writers on the subject of lithotomy, to whom the peculiar instruments as well as the mode of operation formed the chief attraction. The frequency of incontinence of urine in women who have been cut for stone, has induced surgeons to try this as the last resource, and even to practise, when all dilating means have failed, the incision of the mucous lining of the urethra alone.

10. *Aneurism.* The operation for the cure of aneurism was not attempted till 97 A.D., when Rufus of Ephesus tied an artery wounded in venesection, and thus producing aneurism; the artery was, however, divided after the ligature had been applied. Burning, and excision of swellings connected with vessels, were introduced by Celsus, and appear to have been practised till 340 A.D., when Antyllus again introduced the practice of applying ligatures to aneurisms. By this surgeon the artery was laid bare above and below the swelling, and tied in both situations, whilst the aneurismal sac was cleared out, and filled with various substances to produce suppuration. This operation appears to have continued some time, with this exception that after the application of the ligatures the sac was occasionally removed in toto, or destroyed by caustic or actual cautery. The introduction of the tourniquet by Morel, in 1674, was found to be a great advantage in the operation for aneurism, which even at the period of Dionis, Le Dran, and Bertrandi, consisted in laying open the sac and applying a ligature above and below. The plan of opening the sac, and tying the artery above and below, continued even down to the time of John Hunter, with one exception, which does not, however, appear to have attracted much notice. Anel, in 1740, in a case of aneurism at the bend of the arm, cut carefully down to the artery, and after separating the nerve, tied the artery above the sac without opening this, and cured his patient. This operation met with little favour, and was not much noticed; it however may justly be considered as the greatest improvement in the operation on aneurism down to the time of Hunter. In the imperfect condition of surgery the introduction of Anel's method was a great improvement, but in reality the value of this improvement depends more on the bad condition of surgery at the time, than the excellence of the operation itself. In cases of aneurism from disease of the artery, the application of a ligature according to Anel's method would be attended with danger, and often inapplicable, from the diseased condition of the artery, and its benefit would be confined chiefly to aneurism arising from wounds, and principally to those in which the effusion of blood had attained a circumscribed form, and had not been loosely extravasated. The chief improvement in the operation for aneurism is certainly the application of the ligature according to the plan of Mr. Hunter, which consists in tying the artery at a distance from the aneurism. This great discovery, the value of which has been appreciated more and more ever since it was made, is grounded on the three facts, that by this operation the arteries will maintain the life of the limb, that the aneurism will not continue to increase, and that after certain changes it will be removed; but its value is increased by its enabling the surgeon to tie the artery in a convenient position and healthy situation, as well as to avoid all the dangers of the old operation. The merit of this operation belongs in all respects to Hunter, without diminution from any quarter.

In the middle of the eighteenth century Brasdor introduced the plan of tying the artery on the distal side of the aneurism; this operation is however best known in this country by the labours of Mr. Wardrop, as well as by the principal modern surgical works. This operation presents itself as a last resource, when, from the situation of the aneurism, the artery cannot be tied in the common situation; but even in such cases the chances of success are but slight, as in the majority the aneurisms to which it is applicable are of the most incurable kind, and in the large vessels near the heart.

The attention of surgeons in this country and abroad has been directed especially to the subject of aneurism, with the most decided benefit, and with a remarkable absence of all controversy. The knowledge of the capabilities of small arteries to maintain the circulation of the limb has reduced many arteries to the domain of surgery which were supposed to be as far beyond reach by operation as the aorta itself. The knowledge of the action of the ligature, as first completely and clearly shown by Dr. Jones, and since illustrated by Manec, Lawrence, and Travers, has rendered the application of the ligature more certain, and its action better understood, whilst the application of the discoveries of Laennec, and the improvements of them introduced by successive physicians in this country and abroad, has placed a means of accurate and minute diagnosis in our hands, of which our forefathers had not the least conception.

Within the last few years the torsion of arteries has been introduced by Amussat of Paris; and this plan, consisting in separating the internal membrane of the artery from the middle coat and squeezing it back into the artery, whilst the part on the distal side of this portion of retracted membrane contracts forcibly, has been extensively practised on the continent. In this country it has however met with little encouragement, partly because we are always inclined to follow any safe plan of proceeding, to which we have been educated, as well as because this plan is just as difficult as the application of the ligature, without possessing its certainty.

11. *Amputation.* Removal of the limbs by operation was practised at a very early period, and appears till the time of Ambrose Paré to have been a very fatal proceeding. The danger of bleeding appears justly to have occupied all their thoughts, so that very little attention was paid to the formation of the stump. The bleeding was checked by hot irons, compresses, and the most varied means, and yet with all this apparatus, so small was the success of the operation, that one can well agree with the quaint yet mournful maxim of Guy de Chauliac, that it was better to let the limb drop off than to cut it off, for even if the patient gets well after the amputation, he thinks that the limb might have been saved. The process followed by this surgeon consisted in passing pitch-plasters very tight round the joint, and thus causing the limb to mortify; whilst Botalli, a few years afterwards, fell into the opposite extreme, and invented a kind of guillotine for cutting off limbs.

The whole practice of surgery was, however, destined to undergo a marked change by Ambrose Paré, and in no respect more than in the treatment of vessels wounded in amputation. To this great man we are indebted for the application of the ligature as a means of arresting hemorrhage, it might almost be said the discovery of its application, but the honesty of Ambrose Paré induced him to show that, although in practice he might be the inventor, yet that the suggestions for its use were contained in the works of others. The ligature was applied by Paré to wounds of arteries and veins with the best results; yet the value of it was not estimated by many of his own day, and especially the professors of Paris. These revilers, however, were answered in a manner, which they little expected, by Ambrose Paré, who added an apology to his works, in which he detailed a series of successful cases, when the ligature had been employed by him, and pointed out to them suggestions in Avicenna, Vesalius, Johannes de Vigo, Andreas da Croce, and others, of the value of that means which they had abused. Since this time the ligature has gained general use, and its action been completely illustrated by the labours of Morand and Petit in France, and more especially by our own countryman, Dr. Jones. The tourniquet of Morel appears to have consisted of a cord tightened by a stick, whilst a modification of the present common tourniquet was introduced by Petit, which has been gradually improved by the instrument makers to its present condition.

Within the last few years the attention of surgeons has been directed chiefly to the compression of arteries in difficult situations and without exerting pressure on the neighbouring parts. For this purpose, M. Malapert introduced an instrument, which having a fixed point at the back of the neck and curved parts passing round one side of the neck, would enable the surgeon to compress the carotid artery; whilst an instrument having a fixed point at the back of the shoulder was not long since presented to the French Academy for compressing the subclavian artery. The ingenuity and neatness of these instruments are admirable, but the occasions for their use are rare, and even then it may be doubted, whether the thumb of the surgeon is not at least a more useful, and certainly a more simple instrument. The tourniquets of Dupuytren and Signoroni are however well deserving of notice. That of Dupuytren consists of two steel arcs passing on each other so as to form a semicircle, but capable of forming a larger part of a circle, if required; to one extremity a pad is fixed, whilst to the other a pad elevated and depressed by a screw, is applied and capable of being pressed on the artery. The instrument of Signoroni is formed of two steel arcs, moved like a pair of compasses at their junction with a screw, whilst to each end a pad is fixed. Both these instruments are extremely useful, both avoid the surrounding parts and exercise firm pressure on the artery, but whilst the instrument of Dupuytren is capable of the most accurate adjustment, that of Signoroni is less liable to any accidental derangement.

The introduction of the flap amputation by Lowdham, together with the use of the ligature immediately after amputation, as well as the recommendation of the union by the first intention at the end of the seventeenth century, do not appear to have produced much effect, and to have been soon forgotten. The simple flaps of Verdouin and others, were rather made as a preventive against hemorrhage by being firmly fixed against the face of the stump, than as a means of forming a good basis of support, and the flap amputation appears to have been chiefly of the double flap kind at this early period, when the formation of the flaps had reference to the formation of the stump. The proceedings of

Vermale and Ravaton resembled to a certain degree the modern double-flap amputation, but whilst those of the former were rather short, being only about three inches long, those of the latter were unnecessarily bulky, as the amputation was performed by a circular incision down to the bone, and then by cutting a front and back flap by piercing the limb close to the bone, and cutting at right angles to the first incision. The flap amputation arrived very slowly at any degree of perfection, and was indeed perfected at a much later period than the circular mode. The circular mode of Louis which consisted in first dividing the skin and superficial muscles, followed by a higher incision of the deep muscles of the part, as well as the circular mode of dividing the skin and subjacent cellular tissue, and then dividing the muscles introduced by Cheselden and Pott, enabled them to gain a good covering to the bone, and to make a good stump in many cases; these plans were not, however, so likely to attain the perfect circular stump, which Mr. Alanson and Mr. Hey in England, and Richter in Germany, all strove to obtain by different means.

The peculiarity of Mr. Alanson's operation consisted in the division of the skin by a circular incision, followed by the incision of the muscles in a conical form, the apex of the cone being formed by the cut bone and situated at least three inches higher than the incision of the skin.

Mr. Hey and Professor Richter employed the circular incision of the integuments followed by an incision of the superficial muscles, and subsequently by an incision of the muscles situated near the bone.

In these two modes, and more especially in that of Mr. Alanson, the bone was divided high up, and well covered subsequently by the skin, and would produce a good stump. Great, however, as is the merit of Mr. Alanson's mode of amputation, his fame is not that only of a great operator but of an excellent surgeon; the mode of amputation is but a part of his treatment of these cases to which the accurate union of the stump, and the most careful subsequent treatment were also added, equal to the most improved surgery of the present day, and when it is not very well practised, immeasurably superior to it.

The flap amputations, though practised more or less for many years, have been chiefly introduced into London from the continent and Scotland. Although Benjamin Bell and Sir Charles Bell usually performed the circular operation, the double-flap operation has been practised by the officers of the Royal Infirmary of Edinburgh for some years, and has met with the approbation of Sir George Ballingall, Messrs. Syme and Lizars, and has been practised extensively by Professors Liston and Fergusson. Limbs have been cut off in the most varied manner now for many hundred years, yet it still remains undecided whether the flap or circular amputation is the best; but the experience of civil and military surgeons has decided that a good end may frequently be obtained in either way. The excellence of the stump is by all allowed to consist in a complete absence of pain and a perfect use of the remaining portion of limb, whilst the bone is free and covered with a good cushion marked by a narrow cicatrix; and such excellence the experience of Alanson and of the surgeons of Edinburgh have shown to be attainable in either way. But to men with daily experience in hospitals much is comparatively easy, which to the great majority must from limited experience be extremely difficult. The increase of the flap amputations within the last few years is to a certain degree an evidence of their success, as well as of the want of any objection against it, whilst the facility of performance, though not very great, is not so difficult to acquire as to form any just objection against them. One objection alone, the occurrence of secondary hemorrhage, has been urged against them; but the daily experience of surgeons has now removed this imaginary fear. Although the circular operation in good hands is capable of the greatest perfection, yet its perfection is not entirely dependent on the surgeon. When all proceeds well, the surgeon has nothing to regret; but let the stump inflame and suppurate, and a little bare piece of bone remain raw on the face of the stump, and then the subsequent misery of the patient is excessive, and may even justify a second operation. To all, even the most experienced, the amputation of a limb is a matter requiring great accuracy; but to one whose opportunities are limited, it is a matter of the most serious consideration: the difficulties on either side are great, but the tendency during the last few years has been to extend the flap operation, and apparently with an increase of credit to the surgeon and with relief and subsequent comfort to the patient.

12. *Subcutaneous division of muscles and tendons.* This operation does not appear to have been performed till the end of the seventeenth century, when Roonhuysen and Meeckren of Amsterdam divided the sterno-mastoid muscle in two cases of crooked neck, by exposing it and cutting it with a knife or scissors; this operation was also repeated by Gerrhard Ten Haaf of Rotterdam, and Sharp and Cheselden in this country: but previously to the year 1784, the only other muscle divided by exposure and incision was the scalenus of the left side, which was exposed by caustic, and then divided with the knife by one Minnius, probably of Amsterdam, about the year 1738. In the year 1784 the tendo achillis was divided in a case of varus, under the directions of Thilenius of Lauterbach, with success; this operation was repeated by Sartorius of Hachenburg in 1806, on a case of club-foot, with very indifferent success, both operators making a considerable wound in the skin, and the latter also using extreme violence to bring the foot to a proper position.

The incision of the tendons of the knee, fingers, and feet was practised by Michaelis, professor at Marburg, in 1809, with imperfect success; the incision of the tendon being intended to be only partial and being accompanied with exposure.

We now come to the labours of Delpech on this subject, and find that he published in 1823 a case of a boy cured of pes equinus by division of the tendo achillis as early as 1816; this case was, however, attended with very serious illness, and appears to have been the only one operated on by Delpech; but yet the attention of this eminent surgeon was drawn forcibly to this subject, as is shown by his later work.

Delpech, in his "Orthomorphie," published at Paris in 1828, speaks of the division of the tendo achillis as an operation not yet submitted to regular rules or methodically set forth, but as a useful resource; whilst he recommends as rules for the division of the tendon, that it should not be exposed but reached circuitously; that extension should not be tried at first; that extension of the means of union should be subsequently made, and the limb fixed in the new position, until the new tissue is firm. Such are the rules given by Delpech, and which form even now the basis of treatment, whilst the veterinary surgeons operated with the greatest success in France and Germany on the deformed feet of horses and mules, and even prefaced their remarks in one paper by saying that they wished to render an operation generally known, which might perhaps be introduced into human surgery.*

Thus as early as 1828 the remedial means of curing many distortions had been shown in animals and in some few men, whilst the principles of the operation had been well laid down by Delpech; yet no general notice was taken of it; and even in 1835 (one year after the publication of Stromeyer's cases), Dr. Little at Leyden, Leipsic, Dresden, and Berlin, "in answer to his inquiries respecting the operation of division of the tendo achillis, experienced only disappointment." The skill of Stromeyer, however, enabled him to add the case of Dr. Little to his cures, who returning to Berlin, conveyed to Dieffenbach a certain proof of the ability of the present professor of Erlangen.

To Stromeyer is due the great merit of having first set the division of tendons by subcutaneous incision in a practical point of view before the profession; others had performed operations and detailed proceedings which collectively amounted to the same result; but his operation includes all the best points of his predecessors, with the separation of the dangers and the recommendation of a proper plan of treatment. In a short period after the introduction of the operation by him, we may enumerate Dieffenbach at Berlin and Dr. Little in London, who together treated by divisions of tendons the various forms of distortion of the feet, fingers, and neck, as well as contractions of joints, whilst Dieffenbach extended the operation to the cure of old dislocations, curvature of spine, and neck.

Although some may attribute the introduction of the operation for the cure of strabismus to an itinerant surgeon of Rouen, or an unknown surgeon in Italy, or find some slight priority in the operations of others, to Dieffenbach belongs the credit of making the division of the ocular muscles a regular surgical operation, the date of his first operation being December, 1839, just two months subsequent to the first operation of Cunier in France. Since this period the division of the ocular muscles has been practised and recommended by the most eminent surgeons in this country and on the continent. In Germany we may name Stromeyer and Dieffenbach, the introducers of this plan of operation, with Von Ammon, Fricke, Von Wattmann, Chelius, and Carus, whilst in France amongst the supporters of this operation are Velpeau, Jules Guerin, Bouvier, Roux, Duval, and the real original operator Cunier. In England the works of Mr. Lawrence and Mr. Lucas contain a complete summary of this operation, whilst the essays and cases of Messrs. Liston, Lizars, Mackenzie, &c. have contributed to its extension and improvement. The capitals of Russia and Sweden have had supporters of this new operation, and Dr. Hebra speaks with enthusiasm of the success of Diffenbach's pupils on the banks of the Ohio and Mississippi. The division of the recti muscles has been performed in cases of deficient vision and amaurosis, by Mr. Phillips of Brussels, for which latter affection Mr. Adams has also employed the same means; the success of this operation has, however,

* Journal pratique de Médecine Vétérinaire, 1826, p. 202.

not been sufficiently great or marked to induce the repetition of it by others, or at least to lead them to publish their results.

The division of the various muscles of the tongue was suggested soon after the introduction of "tenotomy" as a remedy for stammering, and practised at one time rather extensively; this operation is now rarely heard of, and appears either to have ceased to be a novelty or to have been merely at best a temporary means of relief; probably *at best* the latter. The incisions made by Velpeau, Amussat, Phillips, Baudens, and Bonnet appear to have chiefly acted on the genio-glossus, whilst those of Dieffenbach removed a large part of the tongue, and divided the lingual nerve and ranine artery. If these operations are even free from danger, they require for their performance some proof that the obstacle is in the tongue and remediable by this operation; but as they are not all free from danger and do not necessarily improve the patient, their rash performance is inexcusable in the highest degree.

---

